# The Psychological and Behavioral Patterns of Online Psychological Help-Seekers before and during COVID-19 Pandemic: A Text Mining-Based Longitudinal Ecological Study

**DOI:** 10.3390/ijerph182111525

**Published:** 2021-11-02

**Authors:** Yinghui Huang, Hui Liu, Lin Zhang, Shen Li, Weijun Wang, Zhihong Ren, Zongkui Zhou, Xueyao Ma

**Affiliations:** 1 Key Laboratory of Adolescent Cyberpsychology and Behavior, Ministry of Education, Wuhan 430056, China; yhhuang@ccnu.edu.cn (Y.H.); huiliu931031@gmail.com (H.L.); blue.she.n@163.com (S.L.); wangwj@mail.ccnu.edu.cn (W.W.); ren@ccnu.edu.cn (Z.R.); zhouzk@mail.ccnu.edu.cn (Z.Z.); 2 Key Laboratory of Human Development and Mental Health of Hubei Province, Wuhan 430056, China; 3 School of Psychology, Central China Normal University, Wuhan 430056, China; 4 Medical Psychology, Department of Psychosomatic Medicine and Psychotherapy, University of Ulm, 89075 Ulm, Germany; xueyao.ma@uni-ulm.de

**Keywords:** COVID-19, online psychological help-seeking behavior, online mental health service, text mining

## Abstract

Online mental health service (OMHS) platforms have contributed significantly to the public’s mental health during the COVID-19 pandemic in China. However, it remains unclear why the public used OMHS platforms for psychological help-seeking (PHS) behavior and how PHS behavior varied across different stages of the COVID-19 pandemic. Based on the ecological PHS behavior data from two OMHS platforms, we extracted population, psychological problems, and influential factors of PHS behavior by text mining and time series analysis methods. Seven top-ranked psychological problems (i.e., depression and anxiety, lack of interest, suicidal tendencies, social phobia, feelings of being worried and afraid, suffering, anger) and seven influential factors (i.e., interpersonal relationships, love, family, work, psychotherapy, personal characteristics, marriage) were found. The online PHS behaviors related to different psychological problems and influential factors remained a growing trend before 2020 and have been increasing significantly due to the COVID-19 outbreak. Four main stages were found during the pandemic according to the changes in the online PHS population: sharp growth, significant decline, slight rebound, and slow decline. This study identified large-scale, spontaneous PHS behaviors among the online public during the COVID-19 pandemic and the various psychological problems and influential factors that varied across different stages of the pandemic, suggesting that the government and health practitioners should adopt effective policies and strategies to prevent and intervene in mental health problems for the online public.

## 1. Introduction

Coronavirus disease 2019 (COVID-19) has caused more than 195 million cases and 4.18 million deaths worldwide to date [1]. The Chinese government has implemented sustainable prevention and control measures in response to the COVID-19 pandemic. Although COVID-19 is current well controlled in China, confirmed infected cases have been emerging sporadically. Notably, the pandemic has not only impaired physical health but has also resulted in short-term or long-term psychological problems, especially for people with autoimmune disorders and chronic diseases. According to a review on the prevalence of psychological problems among the general population during the COVID-19 pandemic, the prevalence of stress, anxiety, and depression were 29.6% (95% confidence interval (95% CI): 24.3–35.4), 31.9% (95% CI: 27.5–36.7), and 33.7% (95% CI: 27.5–40.6), respectively [2]. People with autoimmune diseases showed a more than a 2–4 fold increase in the risk of psychological problems during the COVID-19 pandemic [3,4].

Facing the mental illnesses and psychological problems of the general public, online mental health services (OMHS) have become an alternative to offline services in the special context of the COVID-19 pandemic. OMHS are conducive to saving time. More importantly, they have the advantage of allowing the patient and the practitioner to avoid face-to-face contact, which is critical to successfully curbing the spread of the coronavirus [5]. Moreover, a systematic review and meta-analysis investigated the prevalence of anxiety, depression, sleep problems, acute stress symptoms, somatic symptoms, fear, and obsessive compulsive symptoms during different periods of the pandemic in China and found that the prevalence of mental illnesses and psychological problems may have varied during different periods of the pandemic [6]. Investigating the behavioral and psychological impact of the COVID-19 on online psychological help-seekers before and during the pandemic is crucial for the government, researchers, and practitioners to adopt strategies to prevent and intervene in these problems on time.

There are two kinds of methods that can be used to investigate the impact of COVID-19 on the public’s mental health and that have been used in existing studies. The first method is the questionnaire survey. All of the studies and systematic reviews and meta-analyses mentioned above used self-report questionnaires [2,6]. Despite most self-report questionnaires used in these studies being standardized, easy to conduct, and having good reliability and validity, we cannot ignore the potential problems of using this method. For example, most questionnaires or scales measure the symptoms but overlook certain development problems. Data collected by questionnaires or scales reflect the status of the participant at a static point but do not consider the temporal dynamics. More importantly, it is hard to avoid social desirability and memory bias [7].

The second approach is text mining technology. The majority of previous studies crawled posts from social media platforms, e.g., Tweets and Weibo, then utilized sentiment analysis or topic modeling to explore the emotions and topics that appeared in the posts [8,9,10,11]. It is an ecological approach; that is, the data can reflect the temporal dynamics of psychological problems in real-time and over a long period, which may compensate for the deficiencies of questionnaire surveys. However, because of the most previous studies crawled texts from social media platforms, the findings of these studies have several limitations. First, some certain groups of the general public like to use social media platforms to express their opinions, while others are less likely to use them or to express opinions through these platforms. Therefore, existing studies based on these data can only investigate the opinions and attitudes of individuals who actively use social media platforms. Second, the contents of the posts on social media platforms cover a wide range of fields in our lives. Existing studies were only investigating opinions and attitudes about users’ daily lives instead of investigating the temporal dynamics of mental illnesses and psychological problems.

Online platforms specific to PHS have contributed significantly to the COVID-19 pandemic in China and are more suitable than social media platforms for the exploration of the temporal dynamics of mental illnesses and psychological problems during the COVID-19 pandemic. These platforms can be roughly divided into two types, both of which could be complementary to each other: (1) OMHS platforms that were active before the emergence of the COVID-19. Researchers can compare PHS behavior, psychological symptoms, etc., before, during, and after the COVID-19 pandemic by using the data from these platforms. (2) OMHS platforms that were established to specifically deal with the pandemic, e.g., the MOE-CCNU Mental Health Service Platform [12]. On these platforms, most help-seekers were seeking help for their mental illnesses or psychological problems that were related to the COVID-19 pandemic.

The above studies suggest that there are few studies on the psychological and behavioral patterns of online psychological help seekers using OMHS before and during the pandemic. Therefore, one research question being asked during the present research is to understand the reasons why online psychological help-seekers conduct PHS behavior and its patterns before and during the COVID-19 pandemic. Another research question that will be considered in the present research is the examination of the extent to which the temporal patterns between the COVID-19 pandemic and the behavior of online psychological help-seekers concerning psychological problems interact with each other. Specifically, previous studies focused on the psychological impact of the public in the early stages of the COVID-19 pandemic [13,14], while the present study grasped the impact over a longer period (i.e., before and during the pandemic). Moreover, a majority of previous studies focused on the mental status of social media users, while the present study concerned online psychological help-seekers. In general, the present study aims to employ a new approach with more ecological data that minimizes problems such as ignorance and social desirability and grasps a longer period of COVID-19′s impact on online PHS behavior to facilitate the understanding of the longitudinal impact of COVID-19 on the population, psychological problems, and influential factors of the online public in OMHS platforms. The PHS behavior mentioned in the present study includes the population, psychological problems, and influential factors related to online psychological help-seekers using OMHS platforms.

## 2. Materials and Methods

### 2.1. Data Crawling

The first data source is a famous Chinese online counseling platform “One Psychology” (https://www.xinli001.com/, accessed on 30 July 2021), on which nearly 20 million have asked for psychological help. In the Q&A section of the platform, psychological help-seekers are able to post about their psychological distress and problems and are able to seek psychological services and support from the platform’s psychological counselors anonymously. The post may include several optional components such as the title of the post, age, gender of the help-seeker, course of the psychological problem, inner feelings, duration of the problem, and the label (i.e., occupation, marriage, romantic relationship, family, etc.). We utilized “Bazhuayu” (https://www.bazhuayu.com/, accessed on 1 August 2021), a web scraping software, to crawl 54,797 psychological help-seeking questions, of which 3263 posts referred to the COVID-19 pandemic. It was possible for each post to contain the following three components: the title of the description, the description of the psychological problem, and the asking time.

The second data source is the official website of the National Health Commission, on which one could search the number of COVID-19 cases in China, including cumulative confirmed cases, cumulative deaths, new confirmed cases, and new deaths [15].

The third data source is the MOE-CCNU Mental Health Service Platform, which collects time-series data on the number of psychological help-seekers. This platform has been operating since 31 January 2020. Psychological help-seekers can access the platform via WeChat, which is the most popular social network application in China. We collected time-series data on the number of daily psychological help-seekers from 31 January 2020 to 8 January 2021, with a total number of 37,698 psychological help-seekers.

### 2.2. Domain Topic Modeling

For the analysis of the psychological problems and influential factors included in the PHS texts, a neural embedding-based latent semantic analysis method [16] was utilized to extract the domain topics from the texts in the sections for the title and the description of the psychological problem. Neural embedding is a family of techniques that can be used to obtain compact, dense, and continuous vector-space representations of entities that can efficiently encode multifaceted relationships between those entities and has become a core ingredient in modern machine learning [17].

Specifically, first, a predefined lexicon regarding psychological problems and the influential factors concerning mental problems was constructed. The seed words for the lexicon were extracted by two psychology Ph.D. candidates from three text resources: the Kessler 10 and Patient Health Questionnaire [18], the emotional vocabulary from the Dalian Institute of Technology [19], and the question tag system from the One Psychology website.

Secondly, we constructed the domain lexicons for the OMHS communities. Using the Jieba tool (i.e., a Python segmentation package for Chinese) and the Baidu stop-word list, the text from mental health questions was cut, and stop words were deleted. According to the neural embedding-based latent semantic analysis method, the texts were used as the training corpus. To implement this method, we used the word embedding method from the Word2vec in Gensim software (https://radimrehurek.com/gensim/models/word2vec.html, accessed on 1 August 2021) to construct the latent semantic model for large scale PHS texts in order to obtain domain lexicons for psychological problems and related influential factors. Specifically, the cosine similarity between the words in the model vocabulary and the predefined lexicon was calculated based on the model. We recruited two graduate students to set the cosine similarity thresholds to remove words in the PHS texts that were irrelevant to the predefined vocabulary, to check the retained words manually, and then to build the domain lexicons of psychological problems, psychological problems, and influential factors.

Thirdly, we obtained topics concerning psychological problems and influential factors from the help-seekers. According to the domain lexicons, the related words from the help-seekers were selected. Word vector representations of the psychological problems and influential factors were obtained using the average word embedding method [20]. We further used the K-mean clustering algorithm, and its evaluation index (i.e., silhouette coefficient) to evaluate the clustering performance with different numbers of clustering centers [9]. The number of cluster centers under the optimal silhouette coefficient was selected to construct the clusters of psychological problems and influential factors. The silhouette coefficient was calculated by the degree of aggregation and separation of the clusters, and its value ranged from −1 to 1. A higher value represents a better clustering performance. Then, we recruited two Ph.D. candidates to classify the similar symptom manifestations and influential factors topics into several clusters according to high-frequency keywords and to determine the content and number of topics regarding the psychological problems of the help-seekers.

### 2.3. Time Series Analysis

We used time series analysis methods to analyze the longitudinal PHS behavior to obtain the temporal patterns of online PHS behavior before and during the COVID-19 pandemic. The Prophet model is a time series analysis model based on the generalized additive model developed by Facebook. It not only fits the time series data but also monitors the festival effect and trend change nodes. It is capable of generating forecasts of a reasonable quality at scale. According to Taylor and Letham, Prophet always performs better than other approaches [21]. This method allows us to become familiar with the yearly, monthly, and weekly trends of the time series for the PHS population. We further used the annual compound growth rate (CAGR) to quantify the yearly, monthly, and weekly dynamics of the PHS time series. The calculation formula is:

(1) $$M=\sqrt[{n}]{\frac{B}{A}}-1,$$

where *B* is the value from the last year, *A* is the value in the first year, and *n* is the number of years between *A* and *B*. We used the Pearson correlation coefficient to quantify the correlation between the time series of the PHS behavior in OMHS platforms and COVID-19 cases.

**Figure 1:**
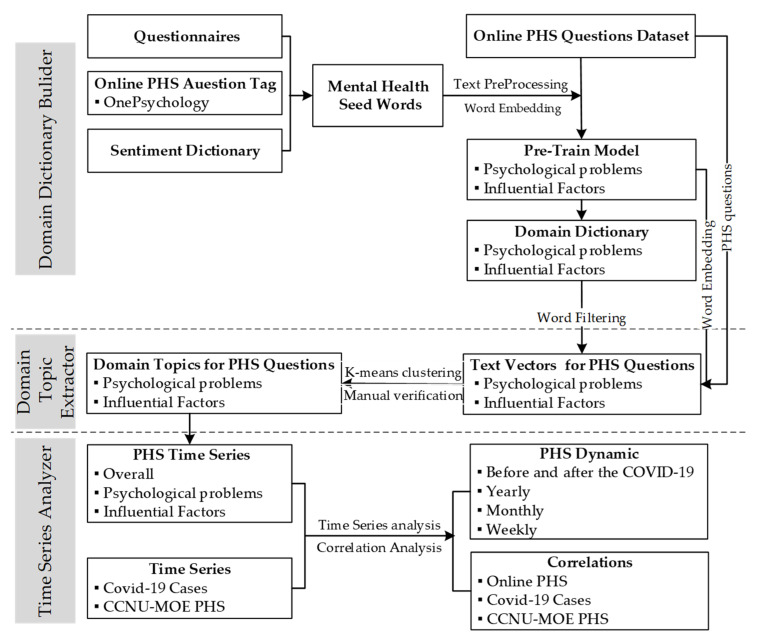
Research methods and processes.

The research methods and processes are shown in Figure 1. Specifically, we first obtained the public’s PHS behavior data using a web crawler, which was mentioned above in the discussion of the OMHS community and the platforms. Second, based on the semantic analysis model, we constructed domain lexicons using the existing knowledge related to mental illnesses and psychological problems. Third, we used the domain lexicons to remove words that were irrelevant to psychological problems and influential factors in the visitors’ questions and obtained the vector representation of every visitor’s questions using the latent semantic model and used the k-means algorithm to cluster the vector representations of all of the visitors’ questions. Then, the typical question clusters and related high-frequency words were identified manually. Fourth, we used the time series analysis method to identify the yearly, monthly and weekly dynamic patterns of PHS behavior and used the correlation analysis method to investigate the relationship between PHS behavior (OMHS community and MOE-CCNU Mental Health Service Platform) and COVID-19 cases.

## 3. Results

### 3.1. Analysis of the Psychological Problems and Influential Factors of the Online PHS Behavior

To understand the psychological needs of online psychological help-seekers and why they ask for help, we extracted seven psychological problems, seven influential factors, and the corresponding keywords (see Table 1 and Table 2). The different psychological problems were related to depression and anxiety, suffering, social phobia, lack of interest, suicidal tendencies, worry (afraid), and anger. The influential factors involved love, marriage, psychotherapy, work, interpersonal relationship, character, and family.

### 3.2. Analysis of the Temporal Changes of Online PHS Behavior before and during the COVID-19 Pandemic

#### 3.2.1. Analysis of the Yearly Trends of Online PHS Behavior before and during the COVID-19 Pandemic

**Figure 2:**
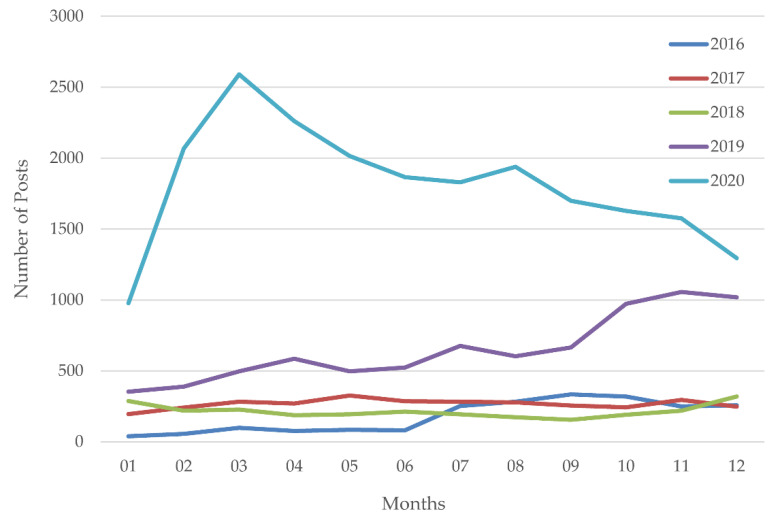
The yearly trends for the PHS posts before and during the COVID-19 pandemic.

To investigate the association between the COVID-19 pandemic and the PHS populations related to different psychological problems and influential factors, we analyzed the general trends of the number of posts made by PHS in the OMHS community from 2016 to 2020 (see Figure 2). In general, the amount of PHS posts showed a slight upward trend in 2016–2018 and an increase in 2018–2019. During the COVID-19 pandemic, the amount increased rapidly. We calculated the CAGR of the PHS posts from 2016 to 2020. Specifically, the CAGR of the PHS posts in the OMHS community was only 54% from 2016 to 2019, while the CAGR of the PHS posts was 179% from 2019 to 2020. The CAGR in the COVID-19 pandemic was about 3.315 (179%/54%) times that of the previous level.

**Figure 3:**
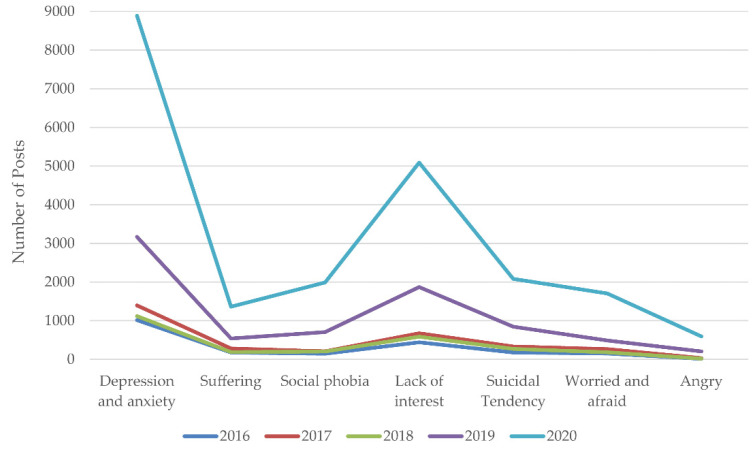
The yearly trends of posts related to different psychological problems before and during the COVID-19 pandemic.

**Figure 4:**
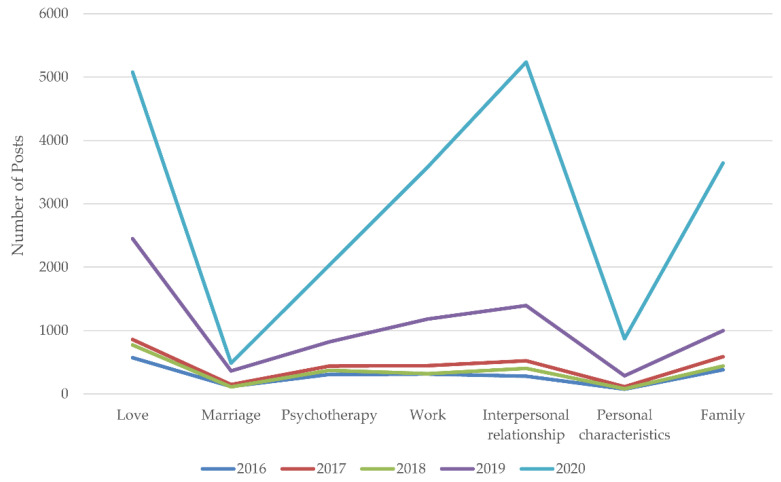
The yearly trends of posts related to different influential factors before and during the COVID-19 pandemic.

We analyzed the overall trends of the PHS population across different topics from 2016 to 2020 (see Figure 3 and Figure 4). Figure 3 shows the overall trends in the number of help-seekers for different types of psychological problems. Specifically, from 2016 to 2019, the CAGRs of depression and anxiety, suffering, social phobia, lack of interest, suicidal tendencies, fear, and anger were 46.12%, 44.67%, 68.56%, 61.76%, 67.75%, 48.07%, and 145.05%, respectively. From 2019 to 2020, the CAGRs for depression and anxiety, suffering, social phobia, lack of interest, suicidal tendencies, fear, and anger were 180.47%, 152.88%, 182.81%, 172.1%, 146.75%, 250.1%, and 188.35%, respectively. The results show that the CAGR remained at a high level, especially in terms of developmental adaptive problems such as fear, anger, and social phobia. Moreover, during the COVID-19 pandemic, the CAGR of psychological problems increased significantly, ranging from 146.75% to 250.1%, which was about 3.17 times (the sum of CAGRs in 2020 divided by the sum of GAGRs in 2016–2019; the calculation method is the same as below) the previous level on average. In particular, the increases in suicidal tendencies and depression were sharp, which were about 4.82 and 4.24 times the previous level.

Figure 4 shows the yearly trends of the posts related to influential factors. Specifically, from 2016 to 2019, the CAGRs of love, marriage, psychotherapy, work, interpersonal interaction, personal characteristics, and family were 62.47%, 45.98%, 38.6%, 55.77%, 70.79%, 56.23%, and 37.73%, respectively. From 2019 to 2020, the CAGRs of love, marriage, psychotherapy, work, interpersonal interaction, character, and family were 107.31%, 33.52%, 147.8%, 202.62%, 275.34%, 204.55%, and 264.93%, respectively. The results show that the CAGR remained at a high level, especially for love, work, interpersonal interaction, personal characteristics, and family. With the exception of marriage, the CAGRs of other influential factors ranged from 107.31% to 275.34% during the COVID-19 pandemic. The CAGR during the pandemic was about 2.764 times those of the previous level on average. Notably, the increases in work, interpersonal interaction, and personal characteristics were sharp, about 6.508, 3.370, and 2.724 times those of the previous level.

#### 3.2.2. Analysis for Stages of Temporal Dynamics for Online PHS Behavior during the COVID-19 Pandemic

**Figure 5:**
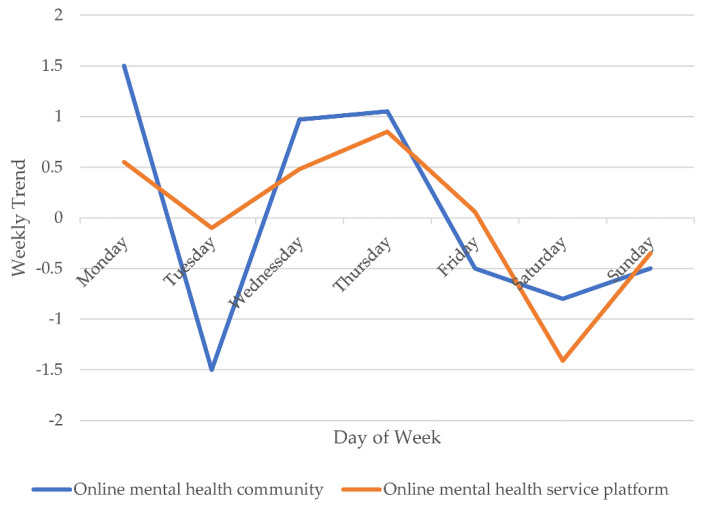
The weekly trends of the number of PHS posts in two OMHS platforms during the COVID-19 pandemic.

**Figure 6:**
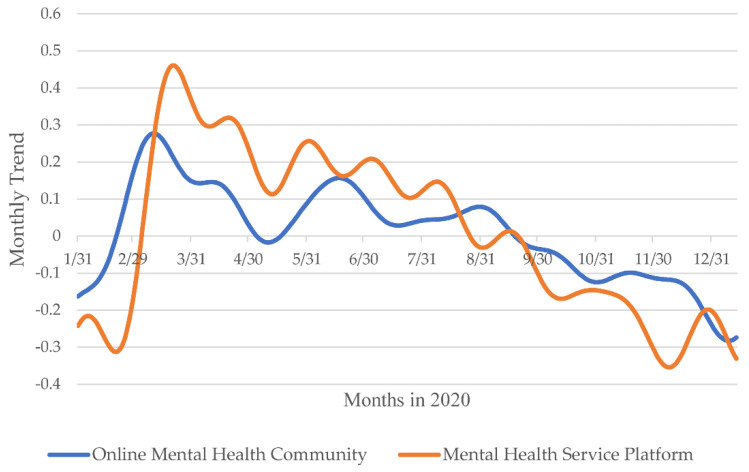
The monthly trends for the number of PHS posts on two OMHS platforms during the COVID-19 pandemic.

To further explore and validate the correlation between the COVID-19 pandemic and the temporal dynamics of the PHS population, we used the time series analysis method to recognize the temporal patterns of the number of PHS posts in the OMHS community and platform during the COVID-19 pandemic (see Figure 5 and Figure 6). Concerning the weekly trends, as shown in Figure 5, two OMHS platforms showed a higher number of posts from PHS on Mondays. In terms of monthly trends, as shown in Figure 6, the dynamics in the number of posts made by PHS on two OMHS platforms can be divided into four stages in 2020: sharp growth (January–March), Significant decline (April–June), slight rebound (July–September), and slow decline (October–December). In addition, there was a positive correlation for the real number of PHS posts between the two OMHS platforms (r = 0.455, *N* = 331, *p* < 0.01). There was a positive relationship between the monthly trends of PHS behavior in the OMHS community and those on the MOE-CCNU Mental Health Service Platform (r = 0.793, *N* = 331, *p* < 0.05).

To validate the close relationship between the pandemic and the temporal dynamics of the PHS population, we further conducted correlation analysis of the temporal patterns between the COVID-19 cases and online PHS posts across different stages. Table 3 shows the relationship between COVID-19 cases and the number of posts due to different psychological problems. During the sharp growth period, the number of posts was positively correlated with the cumulative number of confirmed cases and the number of cumulative deaths (r = 0.774, 0.764, *p* < 0.001), while the number of posts was negatively correlated with the number of new confirmed cases (r = −0.270, *p* < 0.05). During the significant decline period, the number of posts was positively correlated with new confirmed cases (r = 0.274, *p* < 0.001), while the number of posts was negatively correlated with the cumulative number of confirmed cases (r = −0.252, *p* < 0. 0.05) and the cumulative number of deaths (r = −0.249, *p* < 0. 0.05). During the slight rebound period and the slow decline period, there was no significant correlation between the number of COVID-19 cases and the number of posts. The results show that there were stronger correlations between cumulative data (i.e., cumulative confirmed cases and cumulative deaths) and the number of posts compared to the correlations between new increased data (i.e., new confirmed cases and new deaths) and the number of posts, especially during the early stages of the COVID-19 outbreak.

#### 3.2.3. Analysis of Online PHS Behavior during the COVID-19 Pandemic across Different Stages

**Figure 7:**
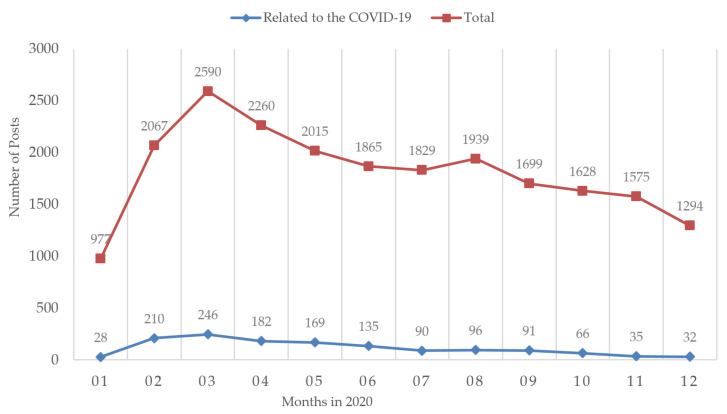
The monthly trends of the number of PHS posts during the COVID-19 pandemic.

**Figure 8:**
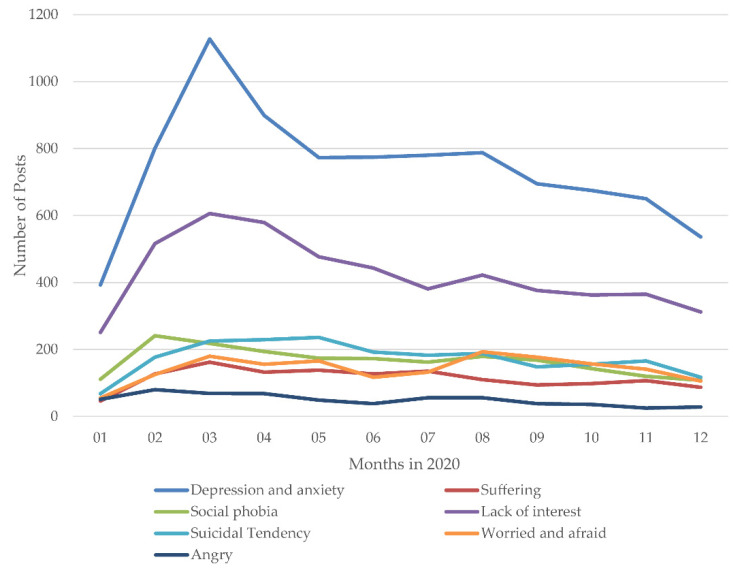
The monthly trends of the number of PHS posts related to psychological problems during the COVID-19 pandemic.

**Figure 9:**
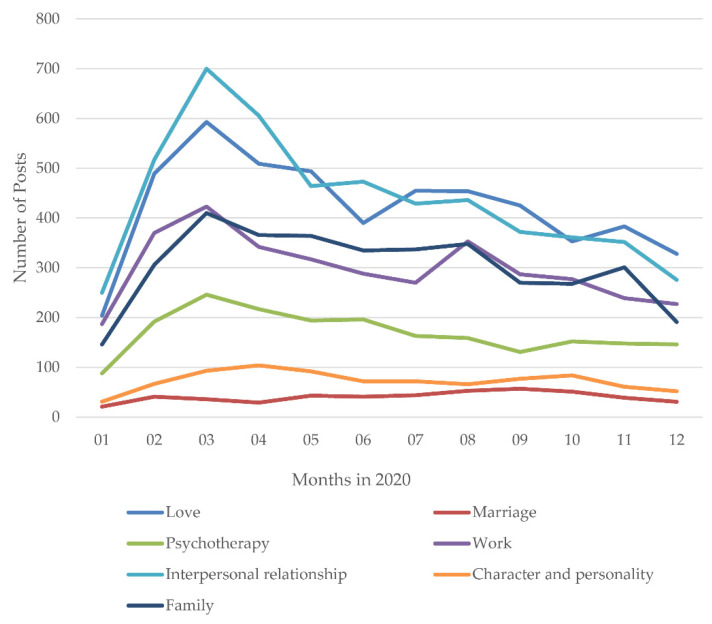
The monthly trends of the number of PHS posts related to influential factors during the COVID-19 pandemic.

To investigate the temporal dynamics of the behavior of the PHS population during the COVID-19 pandemic, we analyzed the monthly patterns of the number of online PHS posts during the pandemic (see Figure 7, Figure 8 and Figure 9). In this analysis, we distinguished posts that explicitly mentioned the COVID-19 pandemic from those that did not explicitly mention the COVID-19 pandemic. As shown in Figure 7, since the outbreak began at the end of January 2020, the number of PHS posts rose rapidly in January and February, culminating in March. In the significant decline period, after a sharp drop in April and May, the number of PHS posts reached a relatively low level in June. In the slight rebound period, the number of PHS posts showed an upward trend and reached a relatively peak in August. In the slow decline period, the number of help-seekers fluctuated slightly and showed a downward trend.

As shown in Figure 8, to investigate the temporal dynamics of the PHS related to psychological problems during the pandemic, we analyzed the monthly trends of the number of PHS posts related to psychological problems across the four periods. Since the Wuhan (the city where the first outbreak occurred in China) lockdown was initiated on 23 January 2020, PHS posts due to depression and anxiety, social phobia, suffering, lack of interest, and anger increased rapidly and reached their peak during the sharp growth period, while suicidal tendencies peaked in the slight rebound period, and feelings of being worried and afraid reached another peak in August. Through the slight rebound and slow decline periods, the number of PHs posts related to different psychological problems gradually decreased to a relatively low level as the pandemic was brought under control. However, the number of PHS posts discussing depression and anxiety, social phobia, lack of interest, suicidal tendencies, and anger reached another relatively high point in the slight rebound period.

As shown in Figure 9, to investigate the temporal dynamics of the PHS behavior related to influential factors during the pandemic, we analyzed the monthly trends of the number of the PHS posts related to influential factors across the four periods. Since the Wuhan lockdown on 23 January 2020, the number of PHS posts related to love, psychotherapy, work, interpersonal relationship, personal characteristics, and family increased rapidly and peaked in the sharp growth period. The number of PHS posts related to psychotherapy and interpersonal relationship reached a relatively high point in the significant decline period. The number of PHS posts related to love, work, interpersonal relationship, and family reached a relatively high point in the slight rebound period. The number of PHS posts related to marriage and personal characteristics reached a relatively high point in September. During the slow decline period, the overall number of PHS posts was at a low level, while the number of help-seekers affected by love and family reached a relatively high point, then declined.

## 4. Discussion

### 4.1. Principal Findings

The present study investigated the population, psychological problems, influential factors, and the temporal patterns of online psychological help-seekers in the context of the COVID-19 pandemic. One of the two major findings concerns what and why online psychological help-seekers conducted PHS behaviors before and during the COVID-19 pandemic. Previous studies found that negative emotions and social interaction problems due to the lockdown were the main issues during the COVID-19 pandemic [2], e.g., a previous study using text analysis on the posts of active Weibo users found that indignation increased after the declaration of COVID-19 in China (i.e., 20 January 2020) [22]. An Italian nationwide survey investigated the negative emotions generated by social distancing during the COVID-19 pandemic and found that the top three emotions were sadness, fear, and anxiety [23]. Besides the known psychological problems and influential factors, the present study revealed a broader and indirect association between the COVID-19 pandemic and online psychological help-seeking behaviors and found that suffering, social phobia, lack of interest, and suicidal tendencies were the main psychological problems faced by online psychological help-seekers during the COVID-19 pandemic, and love, marriage, psychotherapy, personal characteristics, and family were the influential factors of these problems. These psychological problems have also been identified in previous studies as being the main mental health concerns faced by the public during the COVID-19 pandemic. For example, for suffering, lack of interest, and suicidal tendencies, previous research has shown that the COVID-19 pandemic worsened depressive mood and apathy among community-dwelling older adults [24]. As for social phobia, Loades et al. proposed that while it is the associations between social anxiety and social isolation for many children and adolescents are undoubtedly true, it is also worth commenting on the subset of children and youths with social phobia for whom a temporary lessening of distress may be observed while schools are closed, which could be due to a lack of exposure to anxiety-provoking situations in the school environment [25]. Therefore, the increase in social phobia-related PHS behaviors may suggest that for this subset of children and youths, when the time comes for schools to reopen, it may lead to the opposite result. Likewise, for the influential factors related to these problems, some of the most potent risk factors for suicide identified by researchers during the COVID-19 pandemic were social isolation policies, economic downturn due to lockdown [26], widespread social fear, and decreased access to mental health services (e.g., canceled and delayed appointments and treatment) [27]. The implementation and results of these factors will impede people’s lives, and may lead to issues such as detachment from family and friends, shortages of food and medicine, loss of wages, social isolation due to quarantine or other social distancing programs, and school closures [28], which can accelerate mental health difficulties, such as anxiety, depression, and suicidal behavior [28,29]. Moreover, it has been suggested that some personality traits such as neuroticism are associated with the proneness to experiencing negative emotions in response to psychosocial stressors, and who people score high on neuroticism are more likely to experience anxiety, worry, fear, depression, and loneliness [30]. Therefore, personality traits should also be considered when we want to understand the individual responses of PHS during the pandemic.

Considering the temporal patterns of online psychological help-seeking behavior during the COVID-19 pandemic, it is worthy to mention the following findings: First of all, the present study found that online PHS behavior related to different psychological problems and influential factors continued in an early growth trend and increased with the COVID-19 outbreak. The population of people experiencing psychological problems related to PHS behavior due to negative emotions (worry, fear, and anger) was the biggest while severe mental disorders (suicidal tendencies and depression) were the fastest-growing problem. This finding proves that the external environment changed greatly during the COVID-19 pandemic, and the increase of online PHS behavior was a common phenomenon.

Specifically, 1. during the COVID-19 pandemic in 2020, the number of online PHS increased by 179% compared to in 2019, and the GAGR was 3.315 times that during 2016 to 2019. Explicit danger such COVID-19 paired with uncertainty is known to be associated with increased negative emotions and anxiety [31,32]. A survey of Slovak teachers reported an overall increase in negative feelings (e.g., feeling upset, scared, afraid) during the COVID-19 pandemic period [33]. Symptoms such as anxiety, depression, fear, stress, and sleep problems were also seen more frequently during the COVID-19 pandemic [34], and a recent meta-analysis showed the prevalence of anxiety during the COVID-19 pandemic to be almost 32% [2] based on samples from all over the world. Therefore, our findings not only confirmed the findings of previous studies on the huge impact of COVID-19 on the public’s psychological status but also showed that the PHS behavior on the OMHS platforms may be a large-scale spontaneous behavior of the public in response to the psychological crisis; 2. during the COVID-19 pandemic, PHS behavior related to different types of psychological problems and influential factors continued to experience a growth trend before the outbreak and further formed a rapid growth trend. PHS behavior related to psychological problems showed a continuously increasing trend before the outbreak, especially those related to developmental adaptive problems. During the pandemic, the average CAGR of PHS behavior related to different psychological problems was 3.17 times the previous level. One extensive study determined that 0.9% of university students exhibited severe symptoms of anxiety, 2.7% experienced moderate symptoms, and experienced 21.3% mild symptoms [35]. Another systematic review showed the effects that social isolation may have on children and adolescents during the COVID-19 pandemic, with their findings suggesting associations between social anxiety, loneliness, and social isolation [25]. In addition to this, the higher number of developmental and adaptive problem-related PHS behaviors may also be related to OMHS platforms having a higher number of adolescent and young adult users. A report conducted by a famous Chinese OMHS platform “JianDanXinLi” in 2020 (https://www.jiandanxinli.com/public/2020/, accessed on 29 September 2021) reported that visitors in their early adulthood (21–35 years old) accounted for 77.57% of their users. Compared to developmental adaptive problems, the relatively serious psychological problems related to PHS behavior increased faster, reaching more than 4 times those of the previous level, which indicates that some severe mental disorders were triggered by the COVID-19 pandemic [31,32]. One of the possible reasons for such results is that the pandemic has led to a massive public stress response, and another is that China adopted a very strict quarantine policy when the COVID-19 broke out, causing original face-to-face clients to transfer to online psychological service platforms; 3. regarding the influential factors of PHS behavior during the COVID-19 pandemic, work, interpersonal interaction, and personal characteristics related to PHS behavior continued the previously seen large growth trend and formed a sharp growth trend. Previous studies found that the prevention and control measures during the COVID-19 pandemic resulted in mass unemployment and interpersonal stress [36,37]. Our findings confirmed that these negative factors also existed for online psychological help-seekers.

Secondly, the present study found that the dynamics of the online PHS population using OMHS platforms can be divided into four stages by considering the temporal patterns of online PHS during the pandemic: sharp growth, significant decline, slight rebound, and slow decline. 1. According to the five stages of grief, when an individual experiences some inevitable trauma, a sudden and unfortunate blow, or the loss of a loved one, the individual likely expresses denial and anger first, then depression, and finally acceptance [38]. From the perspective of group behavior, we put forward a PHS-related behavioral response pattern of the online public to the COVID-19 pandemic. 2. The present study found monthly and weekly temporal patterns for the amount of PHS behavior. During 2020, the number of PHS behavior was at high levels on Monday, according to the weekly trends, and at high levels in March and September, according to the monthly trends, regardless of the different OMHS platforms. This result could be explained by the following reasons: First, the increase in psychological problems in the early stages of the COVID-19 outbreak and the remission of these problems after the COVID-19 pandemic was controlled could explain why online PHS behavior reached its peak in March. Second, according to the rhythms of our interpersonal lives, Monday is a transition from relaxation to work [39]. It is easy for individuals to distract themselves from work and go to see doctors, which could explain the peak of help-seeking behavior observed on Mondays. Therefore, public health emergencies could affect the mental health of the public and could further influence help-seeking behaviors aiming to solve such psychological problems. In China, the increase of new confirmed cases was the largest in March, and September was the first time that schools resumed classes.

Thirdly, the online PHS population was positively associated with the increased confirmed cases of COVID-19 at the early stages of the pandemic, while it was not significant in the post-pandemic era. These results indicated that the PHS behavior of online psychological help-seekers may have experienced different stages during the COVID-19 pandemic. The present study found that the temporal patterns of different psychological problems and influential factors related to online PHS behavior are different during different stages of the COVID-19 pandemic. Although the PHS behavior mostly reached its peak in the early stage of the pandemic, their temporal patterns are not the same. The number of negative emotions related to PHS behavior peaked in the sharp growth period, to the love, psychotherapy, work, interpersonal relationship, and family, while suicidal intentions and psychotherapy related to online PHS behavior peaked in the significant decline period. When the COVID-19 pandemic was controlled, the number of help-seekers gradually decreased and finally remained stable. During the COVID-19 outbreak, researchers indicated that the mere exposure to information about COVID-19 was associated with higher anxiety levels [40], and symptoms such as anxiety, depression, fear, stress, and sleep problems were seen more frequently [34]. Furthermore, discrimination and stigma related to infectious diseases might make people fearful of infection, which can affect their mental health status [41]. These findings related to psychological distress seen in the public during the COVID-9 outbreak were consistent with the increase in PHS behavior. In addition, researchers demonstrated that prolonged social isolation [42], economic downturn due to lockdown [26], and decreased access to mental health (e.g., canceled and delayed appointments and treatment) [27] were associated with depression, post-traumatic stress disorder, and suicide risk. Therefore, as COVID-19 continues to spread, this finding may be the reason why PHS behavior along with severe psychological problems peaked in the middle and late stages of the pandemic.

### 4.2. Strengths and Limitations

The present study has the following strengths: First of all, this study has extensively studied the population, psychological problems, and influential factors of the PHS behavior of the online public related to the COVID-19 pandemic. As far as we know, this is the first study that has focused on the associations between the COVID-19 pandemic and online psychological help-seeker behavior. Since OMHS platforms have become the main service channel for online psychological help-seekers under the current pandemic, our research has developed previous research results on psychological help-seeking behavior [13,14], helping to facilitate a spectrum of the extensive psychological impact of the COVID-19 pandemic on public mental health.

Secondly, compared to traditional research, the data in this study have the advantages of having good usability and strong authenticity. As for the data usability, this study uses the longitudinal behavior data on online psychological help-seeking using OMHS platforms over five years to support the acquisition of a high-precision timeline for the amount of online psychological help-seeking behavior, psychological problems, and influential factors. Moreover, these ecological data have advantages over traditional questionnaire methods in terms of data scale, acquisition cost, and ease of access. As for the data authenticity, this study ensures the authenticity of the large-scale online PHS data related to the COVID-19 pandemic. On the one hand, previous studies pointed out that questionnaires may cause potential problems such as memory and social desirability bias [43]. On the other hand, social media data mainly concern individual attitudes regarding the COVID-19 pandemic, and research has indicated that individuals tend to conceal their psychological problems on social media [44]. For example, as one respondent in an interview-based study said, “It’s not that I don’t have problems, I’m just not putting them on Facebook” [45]. The present research used anonymous online PHS behavior data, avoiding the potential problems of memory or social desirability bias that can be made apparent by questionnaire.

Thirdly, the domain topic modeling method used in our study has advantages over the classical topic modeling method used to characterize topics in a fine-grained granularity. This study utilized the text topic mining method based on neural embedding, which is able to extract the topics of psychological problems and influential factors; while the traditional topic model method cannot classify and extract heterogeneous topics.

Fourthly, this study deepens our understanding of online PHS behavior in the middle and late stages of the COVID-19 pandemic. The present study considered the COVID-19 pandemic as several development stages, allowing us to provide a full picture of the population, psychological problems, and influential factors of online PHS behavior. This could remedy the shortcomings of most previous studies, which examined mental illness and help-seeking behavior only in the early stages of the COVID-19 outbreak [10,11,22,23,28,34,37,41].

Last but not least, OMHSs have become one of the most important channels that the public uses for psychological help. The present study shows that investigating the psychological problems and influential factors of the online public is an efficient alternative to offline investigation. Therefore, our study validates that OMHSs can not only provide extensive psychological support for the public but that they have also become a window for researchers and organizations to continuously observe, track, and intervene in the mental health status of the public.

The present study is not without limitations. Firstly, culture may affect the impact of the COVID-19 pandemic on emotional expression and issues of concern that are shared by the online public. The present study only investigated Chinese PHS behavior during the COVID-19 pandemic and lacks support for data from different countries and cultures. Hence, these findings should be cautiously interpreted in terms of their generalizability. Future studies should aim to replicate the present findings by comprising different countries and cultures.

Secondly, although the present study aims to investigate the relationship between the COVID-19 pandemic and the online PHS behavior of the public as a whole, there is still a lack of understanding of differences between different ages, genders, and other important variables. As far as we know, psychological help-seekers on all OMHS platforms are anonymous. When they post PHS information, demographic information such as gender, age, and occupation are not required. Although most help seekers do not expose demographic information on the platform explicitly, we can use machine learning technology to predict this information in subsequent studies.

### 4.3. Implications

In general, there are several important implications to understanding PHS behavior during the COVID-19 pandemic in China. One implication is that the changes to a population, psychological problems, and influential factors of online PHS behavior can help psychological counselors and psychotherapists grasp the transformation of help-seekers and develop strategies to service these help-seekers in the context of a major public health emergency. Another implication is that online platforms are important tools for individuals who have mental illness and psychological problems to seek psychological help during the COVID-19 pandemic. Therefore, governments and health systems should increase their attention and provide adequate financial and policy support for the online psychological services during this period.

Specifically, as for online psychological service agencies, they should first focus on the problems of online psychological help-seekers such as depression and anxiety, suffering, social phobia, lack of interest, suicidal tendencies, worry (afraid), and anger, and then cultivate an online psychological assistance force related to these problems and take on targeted interventions for online psychological help-seekers in different stages of the COVID-19 pandemic. That is, in the sharp growth period of the COVID-19 out-break, interventions should focus on stress response and emotional problems, while in the significant decline period, psychotherapy and interventions for severe psychological problems should be added.

As for the government and institutions, they can use online media to raise public awareness for the prevention of psychological problems and guide the public to consciously use online psychological assistance services. In response to influential factors such as love, marriage, psychotherapy, work, interpersonal relationships, personal characteristics, and family, the government should promote the resumption of work and support the opening of psychotherapy institutions to support people in crisis. Meanwhile, the government should help people with psychological distress to repair any interpersonal, marital, and family relationships that were destroyed during the COVID-19 pandemic through schools, universities, and community organizations. Moreover, the government and online psychological services should additionally increase psychological assistance resources related to suicidal tendencies and depression and should develop contingency plans to fully meet the demands of the mass increase of people in crisis during the COVID-19 pandemic. Meanwhile, the government should take administrative measures to support the resumption of work and production, safeguard people’s work, and rebuild their interpersonal interactions. In addition, appropriate publicity strategies should be adopted during the COVID-19 outbreak to avoid potential adverse effects on the public’s mental health by not over-publicizing the cumulative number of cases.

## 5. Conclusions

Overall, the present study investigated online PHS behavior before and during the COVID-19 pandemic in China. Specifically, the present study identified the population, psychological problems, and influential factors related to the temporal patterns of online PHS behavior in different stages of the COVID-19 pandemic and examined the associations between PHS behavior and the increasing number of confirmed COVID-19 cases. We found that PHS behavior increased sharply after the declaration of the COVID-19 outbreak. Negative emotions may be the main issue for help-seekers. At different stages of the COVID-19 pandemic, the types of psychological problems and influential factors varied. Psychological counselors and psychotherapists could use the data presented here to grasp the transformation of such changes to provide targeted and higher quality services in different stages of the COVID-19 pandemic. Governments and public health systems should pay more attention to online psychological assistance in the context of the COVID-19 pandemic.

## Figures and Tables

**Table 1:** Psychological problems related to topics of the online PHS posts.

The Topics of Psychological Problems	High-Frequency Word (Ranking in Order)
Depression and anxiety	depression, anxiety, insomnia, obsessive compulsive disorder, depressive symptoms, diagnosis, bipolar, despair, violence, shadows, trauma, extreme, headaches, waking up, staying up, dreaming
Suffering	unhappy, sad, uncomfortable, wronged, embarrassed
Social phobia	communication, self-abasement, introversion, sensitivity, lack of self-confidence, dissocial, cowardice, dependence, eye contact, avoidance, conversation
Lack of interest	no interest, no drive, no confidence, no enthusiasm, no desire
Suicidal tendencies	suicide, self-harm, tendency, breakdown, fear of pain, despair, escape, regret, torture, bad
Worry, afraid	fear, worry, tension, doubt, struggle, avoidance, rejection, nausea
Angry	anger, dislike, tantrums, bullying, grievance, disgust, blame, rejection, excess, ugliness, grumpiness, dissatisfaction, selfishness, trust, respect

**Table 2:** Influential factors related to topics of the online PHS posts.

The Topics of Influential Factors	High-Frequency Word (Ranking in Order)
Love	love, boyfriend, relationship, girlfriend, heterosexual, confession, break up, good feeling, gay, single, ex, meet, ex-boyfriend, reunion, first love, ex-girlfriend, Cold War, entanglement, long-distance relationship
Marriage	marriage, divorce, children, pregnancy, wife, man, mother-in-law, husband, married, sex, birth, in-laws
Psychotherapy	treatment, diagnosis, pandemic, anxiety, disorder, medication, mental illness, withdrawal, bipolar, character, cognition, character disorder, schizophrenia
Work	job, graduation, resignation, income, economy, pressure, development, unemployment, job-hopping, career, boss
Interpersonal relationship	communication, character, contact, friend, speech, relationship, conversation, eye contact, dealing, indifference, impression, avoidance
Personal characteristics	character, emotion, life, growth, cognition, conflict, obstacle, age, communication, impression, shadow, avoidance, dominance, character disorder
Family	parents, mother, family, mom, father, dad, brother, grandmother, sister, daughter, grandparents

**Table 3:** Correlations between the COVID-19 cases and the number of PHS posts due to different symptoms.

COVID-19 Cases	Periods in 2020	Number of Online PHS
Cumulative confirmed cases	sharp growth	0.774 **
	significant decline	−0.252 *
	slight rebound	
	slow decline	−0.575 **
Cumulative deaths	sharp growth	0.764 **
	significant decline	−0.249 *
	slight rebound	
	slow decline	−0.555 **
New deaths	sharp growth	
	significant decline	
	slight rebound	
	slow decline	−0.301 **
New confirmed cases	sharp growth	−0.270 *
	significant decline	0.274 **
	slight rebound	
	slow decline	−0.435 **

Note. *N* = 59 (month 1–3), *N* = 90 (month 4–6), *N* = 91 (month 7–9), *N* = 91 (month 10–12); * *p* < 0.05, ** *p* < 0.01.

## Data Availability

The data presented in this study are available upon request to the authors. Some variables are restricted to preserve the anonymity of study participants.
